# Adverse Maternal and Neonatal Outcomes Among Women of Advanced Maternal Age in a Tertiary‐Care Setting in Bangladesh: A Cross‐Sectional Study

**DOI:** 10.1002/hsr2.72424

**Published:** 2026-04-22

**Authors:** Humayra Afroz Pinky, MD Nahid Hassan Nishan, Gazi Md. Ehsanul Alam

**Affiliations:** ^1^ Department of Public Health North South University Dhaka Bangladesh; ^2^ Projahnmo Research Foundation Dhaka Bangladesh

**Keywords:** advanced maternal age, Bangladesh, birthweight, neonatal outcomes, prematurity

## Abstract

**Background and Aims:**

Pregnancy at advanced maternal age is increasing globally, yet risks related to hypertensive disorders, prematurity, and impaired fetal growth remain substantial, particularly in tertiary‐care settings. This study aimed to quantify maternal and neonatal complications among women aged ≥ 35 years delivering in tertiary hospitals in Dhaka, Bangladesh, and to examine factors associated with adverse maternal and neonatal outcomes.

**Methods:**

We conducted an analytical cross‐sectional study at discharge in two tertiary referral hospitals. Consecutive eligible women aged ≥ 35 years were enrolled (*N* = 384). Primary outcomes were binary composites of any maternal adverse outcome and any neonatal adverse outcome. Associations were examined using Pearson's *χ*² tests, and adjusted odds ratios (AORs) with 95% confidence intervals were estimated using Firth penalized logistic regression to address sparse data.

**Results:**

Maternal adverse outcomes occurred in 97.7% of women, most commonly preeclampsia (27.3%), premature rupture of membranes (26.0%), and oligohydramnios (23.2%). Neonatal adverse outcomes affected 80.5% of births, dominated by prematurity (72.9%), preterm delivery (74.0%), and low birthweight (52.3%). Very‐low birthweight was markedly more frequent among infants of mothers aged ≥ 40 years than 35–39 years (51.9% vs. 7.6%; *p* < 0.001). In adjusted analyses, no factors remained independently associated with maternal adverse outcomes. For neonatal outcomes, normal birthweight was strongly protective compared with very‐low birthweight (AOR 0.03, 95% CI 0.01–0.35), while grand multiparity was associated with lower odds compared with primiparity (AOR 0.04, 95% CI 0.00–0.86).

**Conclusion:**

In this tertiary referral context, maternal complications were nearly universal, while neonatal risk was strongly structured by birthweight. These findings support age‐attuned antenatal surveillance and preparedness for small‐baby care among women of advanced maternal age.

**Abbreviations**
AMA: advanced maternal age
ANC: antenatal care
AOR: adjusted odds ratio
APH: antepartum hemorrhage
ARDS: acute respiratory distress syndrome
AUC: area under the (ROC) curve
BDT: Bangladeshi Taka
BMI: body mass index
CI: confidence interval
CS: cesarean section
ENAP: every newborn action plan
EPMM: ending preventable maternal mortality
GFF: global financing facility
IRB: institutional review board
KMC: kangaroo mother care
LBW: low birthweight
MAS: meconium aspiration syndrome
NSU: North South University
NVD: normal vaginal delivery
PPH: postpartum hemorrhage
PROM: premature rupture of membranes
ROC: receiver operating characteristic
UNFPA: United Nations Population Fund
UNICEF: United Nations Children's Fund
VLBW: very low birthweight
WHO: World Health Organization

## 1 Introduction

Across health systems, a defining contradiction has emerged: women are giving birth later, while the clinical risks associated with advanced maternal age remain substantial and, in the forties, markedly steep [1]. In most OECD countries, the mean age at childbirth has increased by 2–5 years since the 1970s, driven by expanded education, urban employment, and changing fertility preferences [2, 3]. The clinical consequences of this shift are most visible in tertiary hospitals, where complicated pregnancies concentrate, exposing a growing mismatch between reproductive timing and the configuration of antenatal and intrapartum services [4, 5].

The public‐health stakes are large globally [6]. Preterm birth is the leading direct cause of under‐five mortality worldwide [7]. In 2020, an estimated 13.4 million infants were born preterm, contributing to nearly one million deaths and substantial long‐term morbidity among survivors [8, 9, 10, 11]. South Asia carries some of the highest rates globally, underscoring the scale of preventable loss concentrated in a small number of countries [12, 13]. South Asia bears a disproportionate share of this burden [12, 13]. Hypertensive disorders, central to later‐age pregnancy risk, affect a substantial proportion of pregnancies globally and contribute to both maternal instability and perinatal compromise, despite the availability of effective, low‐cost interventions [14, 15]. Global initiatives such as the Every Newborn Action Plan (ENAP) and the Ending Preventable Maternal Mortality (EPMM) agenda emphasize early risk detection, growth surveillance, and strengthened newborn care, priorities reinforced through financing mechanisms such as the Global Financing Facility (GFF) and recent philanthropic investments [16, 17, 18, 19, 20, 21, 22].

Against this policy backdrop, the scientific literature is clear on direction yet leaves important gaps for practice. Multiple large cohorts and meta‐analyses show that childbearing at older ages is associated with higher odds of pre‐eclampsia, preterm delivery, fetal growth impairment, stillbirth, and cesarean delivery, even after accounting for baseline comorbidities [15, 23, 24]. The gradient is not linear: risk rises through the mid‐to‐late thirties and steepens in the forties, particularly for hypertensive disease and very preterm birth [25, 26]. Professional guidance mirrors this pattern by recommending closer surveillance of blood pressure, glycaemia, and fetal growth as age advances [27].

Referral hospitals routinely receive older gravidae with coexisting hypertension, diabetes, or thyroid disease; they also shoulder the consequences: pre‐eclampsia, oligohydramnios, membrane rupture, prematurity, respiratory distress, and stillbirth within the same clinical episode [28]. For policymakers and clinicians, actionable hospital‐based evidence is needed that distinguishes women in their forties from those in their thirties, aligns analytic variables with routine ward documentation, and applies appropriate methods for sparse data and rare events. Such evidence directly supports ENAP/EPMM priorities and current investment streams focused on maternal risk stratification and small‐baby care [29, 30].

This study addresses these gaps in a high‐volume metropolitan referral setting in Bangladesh. We quantify maternal and neonatal complications among women of advanced age and examine how sociodemographic, clinical, and obstetric factors relate to composite maternal and neonatal adverse outcomes within the same delivery episode, using ward‐standard definitions to preserve one‐to‐one fidelity from chart to analysis. Therefore, this study aimed to describe the burden and clinical patterns of maternal and neonatal complications among women aged ≥ 35 years admitted to tertiary‐care hospitals in Bangladesh, and to examine associations between maternal characteristics and adverse outcomes within this referral population. The novelty of this study lies in three features: the clear separation of women in their forties from those in their thirties to reflect a steeper clinical gradient; and the explicit linkage of maternal conditions, birthweight, and neonatal morbidity at the point of care.

## 2 Methods

### 2.1 Study Design

We conducted an analytical cross‐sectional study. Given the cross‐sectional and hospital‐based design, analyses were intended to identify associations rather than causal effects. All data were collected at discharge using structured interviews verified against inpatient records, partographs, laboratory results, and neonatal charts. This design enabled assessment of maternal and neonatal complications at the point of care, evaluation of their co‐occurrence with prespecified clinical and social factors, and minimization of recall bias and attrition through a single, well‐documented clinical encounter.

### 2.2 Study Population and Place of Study

Participants were postpartum women aged ≥ 35 years who delivered a live birth or stillbirth in two tertiary referral hospitals in Dhaka (Dhaka Medical College Hospital and Shaheed Suhrawardy Medical College and Hospital). These sites were selected a priori because of their high delivery volume and referral mix, comprehensive and standardized clinical documentation enabling record verification, availability of continuous obstetric and neonatal services for contemporaneous outcome ascertainment, and established administrative processes that supported consecutive recruitment with minimal disruption. Although hospital‐based, the referral catchment of these centers ensures relevance to tertiary maternity care in metropolitan Dhaka.

### 2.3 Study Period and Eligibility

Data were collected from August 2024 to February 2025 across two tertiary referral hospitals in Dhaka. Women were eligible at discharge if they were aged ≥ 35 years at delivery, had a live birth or stillbirth during the study period, had sufficiently complete records to classify maternal and neonatal outcomes, and could complete a brief interviewer‐administered questionnaire. Women who declined participation, were clinically unstable at discharge, or had missing key outcome data (e.g., undocumented complications or birthweight) were excluded. Restricting eligibility to the discharge encounter ensured uniform timing of measurement, minimized recall error, and enabled direct verification of outcomes against inpatient and neonatal records, maintaining consistency between data capture, analysis, and reporting.

### 2.4 Sampling Technique and Sample Size

We used consecutive purposive sampling. Every eligible woman in the advanced‐age band (≥ 35 years) was approached in sequence during the study window. This approach was selected because advanced maternal age is less frequent than routine deliveries in high‐throughput tertiary wards; taking all consecutive eligible cases was the most practical way to attain the target sample while avoiding staff‐mediated selection and minimizing missed cases in a fast clinical flow. The a priori minimum required sample was derived using the single‐proportion formula with conservative assumptions to maximize precision when the true prevalence is uncertain:

$$n\;=\;\frac{Z_{0.95}^{2}\;p(1-p)}{d^{2}}\;\text{with}\;Z_{0.95}=1.96,\;p=0.50,\;d=0.05$$

$$n\;=\;\frac{{\left(1.96\right)}^{2}\times 0.50\times 0.50}{{\left(0.05\right)}^{2}}=\frac{3.8416\times 0.25}{0.0025}=384.16\approx 384$$

A total of 392 deliveries were initially recorded. After applying eligibility criteria and removing duplicate entries identified during data verification, 384 observations remained for analysis, corresponding to a retention rate of 97.96% and an exclusion proportion of 2.04%. The final analytic sample of 384 met the pre‐specified minimum sample size and preserved the intended ±5% absolute precision at the 95% confidence level under a conservative variance assumption (*p* ≈ 0.5). Missing data were minimal across study variables. Analyses were conducted using complete‐case estimation, whereby observations with missing values for variables included in a given model were excluded through listwise deletion. The low proportion of missingness and absence of systematic patterns during data checks suggested that complete‐case analysis was unlikely to materially bias estimates; therefore, no imputation procedures were undertaken.

### 2.5 Outcome Measure

Two binary primary outcomes were defined a priori and constructed at discharge from the treating team's diagnoses and newborn charts, then cross‐checked with the interview to ensure one‐to‐one agreement between source records and analytic coding. Any maternal adverse outcome was coded “present” if any of the following appeared in the delivery record: antepartum hemorrhage, hyperemesis gravidarum, premature rupture of membranes, postpartum hemorrhage, pre‐eclampsia, eclampsia, obstructed labor, oligohydramnios, polyhydramnios, puerperal sepsis, or maternal death; absence of all listed events was coded “absent.” Any neonatal adverse outcome was coded “present” if one of the following was documented in neonatal notes: low birthweight, prematurity, preterm delivery, stillbirth, congenital malformation, meconium aspiration syndrome, or acute respiratory distress syndrome. Stillbirths were included in the neonatal composite outcome and retained in neonatal regression analyses. Data came from the inpatient record plus a brief discharge interview; discrepancies were resolved in favor of the chart.

### 2.6 Independent Variables

Covariates were selected based on prior literature, established clinical relevance, and a predefined conceptual framework reflecting evidence‐supported relationships among sociodemographic, clinical, pregnancy‐related, and delivery factors, while remaining consistent with ward documentation to ensure reproducibility and interpretability. The framework guiding covariate selection and model specification is presented in Figure S1. Within this structure, maternal and neonatal outcomes were treated as parallel dependent variables, and birthweight was considered a downstream clinical indicator primarily relevant to neonatal outcomes. Sociodemographic factors included age, which was grouped as 35–39 years (early advanced maternal age) and ≥ 40 years (very advanced maternal age) because ≥ 35 is the widely accepted threshold for advanced maternal age and the study hospitals treat ≥ 40 as a distinct high‐risk band; this mirrors routine ward practice and leaves sufficient numbers in each group for reliable comparisons [31, 32]. Education was recorded as none, primary, secondary, or higher as captured on admission. Household income used ≤ 15,000; 15,001–20,000; > 20,000 BDT. Cut‐points anchor to Bangladesh's household‐level upper poverty line (≤ 15,000), with 20,000 set just above this threshold to distinguish vulnerable from better‐off groups; they also fit the national wage context and match the observed heaping at 15,000/20,000 in our data, improving interpretability. Family support was coded yes/no based on the question: “During this pregnancy, did you live with family and receive regular help (daily tasks, transport in emergencies, accompaniment to antenatal visits, or financial support)?” “Yes” was coded if either co‐residence or routine instrumental support was reported.

Clinical and obstetric variables replicated the chart to preserve fidelity from measurement to analysis. Comorbidity (any/none) was built from a checklist verified against the record (thyroid disease, diabetes, hypertension, anemia, asthma/allergy, infectious disease, endocrine/metabolic, cardiac, other, with “none” recorded separately). Nutritional status was abstracted exactly as recorded in the inpatient chart, where the treating team summarizes it in four categories: poor, moderate, good, and excellent per the hospital's standard clinical assessment; we retained the four labels verbatim for concordance with the source record. Because nutritional status and adiposity are not interchangeable, BMI was analyzed separately using the Asian cut‐offs used in the clinical notes (normal 18.5–22.9; overweight 23.0–27.4; obese ≥ 27.5). Physical activity during pregnancy (sometimes, occasionally, regularly) followed the response options printed in antenatal notes to limit recall ambiguity. Antenatal care (ANC) contacts were grouped as 0–3, 4–7, and ≥ 8 visits; these cut‐points reflect how ANC cards are summarized on the ward and provide clinically meaningful tiers for service exposure [33]. These categories were chosen in accordance with World Health Organization recommendations, which specify a minimum of eight antenatal contacts for optimal care; categories with fewer contacts therefore represent progressively reduced exposure to recommended antenatal services. Parity (1; 2–4; ≥ 5) and gravidity (1–2; 3–4; ≥ 5) used routine obstetric history cut‐points; the ≥ 5 category captures grand multiparity recognized in local practice and avoids small unstable cells. Mode of delivery (normal vaginal delivery, cesarean section), place of delivery (home, hospital; retained to mirror the register), and plurality (singleton, twin) were abstracted directly from delivery records. Birthweight used the ward's three‐band chart: very low birthweight < 1500 g, low birthweight 1500–2499 g, and “normal” 2500–3800 g; we kept the 2500–3800 g upper bound exactly as recorded to avoid post‐hoc recoding that could shift denominators and to maintain one‐to‐one alignment with the results. Sources of data were the discharge interview (demographics, family support, work, activity) and the chart (age/DOB, ANC counts, parity/gravidity, comorbidities, BMI, delivery mode/place, plurality, birthweight); discrepancies were resolved against the chart as the reference.

### 2.7 Data Collection

All measurements were obtained at discharge to ensure uniform timing and complete documentation. Trained public‐health graduates administered brief structured bedside interviews to collect sociodemographic information and self‐reported physical activity, and verified clinical variables directly from inpatient and neonatal records, including antenatal care visits, parity and gravidity, delivery characteristics, birthweight, BMI, and documented maternal and neonatal complications. The data collection tool was piloted and refined, with same‐day checks for completeness and internal consistency; duplicate records were removed during cleaning, resulting in a final analytic sample of 384.

Quality assurance was overseen by senior faculty, who reviewed validation summaries, resolved discrepancies, and approved final coding rules. Additionally, a 2% random sample of records was independently re‐abstracted and cross‐checked against source charts, with discrepancies corrected prior to data lock, ensuring concordance between interview data and clinical records.

### 2.8 Data Analysis

**Figure 1:**
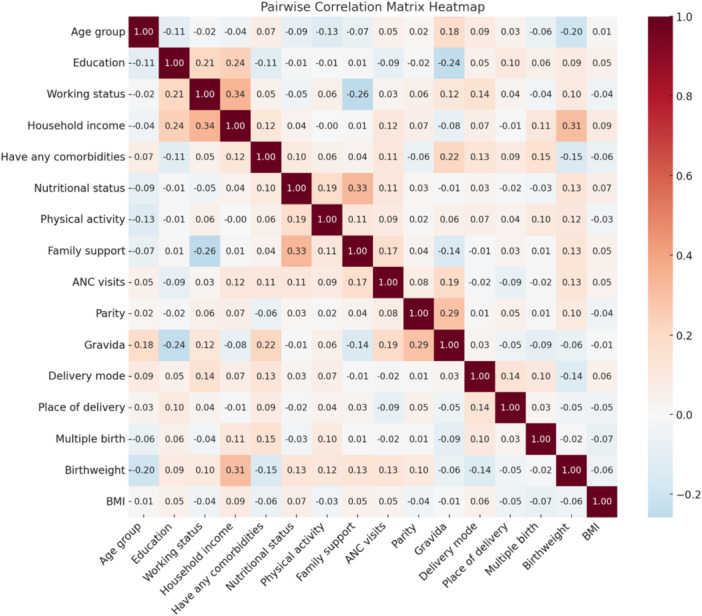
Heatmap showing Pairwise correlation matrix of the independent variables. *Note:* The heatmap shows correlation coefficients among candidate sociodemographic and clinical predictors. Color intensity indicates the strength and direction of association. The matrix was used to assess multicollinearity before multivariable analysis.

All analyses were conducted using Stata version 17 (StataCorp, College Station, TX). Univariate summaries are presented as counts and percentages for all categorical variables to describe the study sample. Bivariate associations between participant characteristics and each composite endpoint were assessed using Pearson's *χ*² tests, applying consistent category boundaries across analyses to ensure comparability. Effect sizes are reported as adjusted odds ratios (AORs) with 95% confidence intervals (CIs) and are the primary measures of association. *p* values are reported as supplementary information and are not interpreted in isolation. All *p* values are two‐sided and reported using standard conventions. Effect sizes (adjusted odds ratios) with 95% confidence intervals are presented as the primary measures of association; *p* values are reported to indicate statistical compatibility with the null hypothesis and are not interpreted in isolation. Before modeling, multicollinearity among candidate predictors was assessed using pairwise Pearson correlation matrices Figure 1. Visual inspection of the correlation heatmap showed no problematic bivariate associations among predictors. To further evaluate multivariable collinearity, Variance Inflation Factors were calculated, yielding a mean VIF of 3.64, with all individual values below the commonly accepted threshold of 10, indicating no evidence of harmful multicollinearity. Slightly elevated values observed among nutritional, anthropometric, and reproductive variables reflected expected clinical covariance rather than statistical instability. Accordingly, all prespecified covariates were retained in multivariable models. For multivariable estimation, we fitted Firth penalized logistic regression models for each endpoint to obtain adjusted odds ratios (AORs) with 95% confidence intervals. This approach was chosen a priori because several cross‐tabulation cells were small or zero (including strata with 100% events), a condition that produces separation and unstable maximum‐likelihood estimates; the penalized‐likelihood method is a recognized remedy in sparse binary‐outcome settings and is widely applied in obstetric outcomes research. Because the maternal composite outcome was nearly universal in this study, resulting in limited outcome variability, adjusted estimates for maternal outcomes are interpreted descriptively rather than as evidence of independent associations. In this context, statistics such as the Wald *χ*² and AUC are presented to characterize model behavior under separation rather than to imply predictive performance. Birthweight was included in the neonatal model as a proximal clinical marker summarizing upstream maternal, placental, and gestational processes rather than as an etiologic exposure. Because low birthweight is part of the neonatal composite outcome, coefficients for birthweight are presented to characterize neonatal vulnerability and referral case‐mix and are interpreted descriptively rather than causally. All statistical tests were two‐sided, with an a priori significance threshold of *α* = 0.05. Statistical reporting follows SAMPL guidelines for biomedical research.

### 2.9 Ethical Considerations

The study was approved by the North South University Institutional Review Board (IRB No. 2025/OR‐NSU/IRB/0810). Provisional approval permitted supervised initiation of enrollment, with final approval granted before completion of data collection and before any analysis. Written informed consent was obtained at discharge after clinical stabilization, and participation was voluntary and did not affect care. Confidentiality was ensured through private interviewing, coded study identifiers, separation of identifiers from research data, and de‐identified aggregate reporting, with files stored in password‐protected, access‐restricted repositories. As a non‐interventional, records‐based postpartum study, risks were limited to confidentiality and were mitigated by these safeguards. All procedures complied with institutional policies and the Declaration of Helsinki.

### 2.10 Result

Table 1 presents the sociodemographic and obstetric characteristics by maternal age. Of 384 women, 27 (7.0%) were aged ≥ 40 years. Compared with women aged 35–39 years, those aged ≥ 40 years differed significantly in physical activity, gravidity, and birthweight (all *p* < 0.001). Very‐low birthweight (< 1500 g) was substantially more common among infants of women aged ≥ 40 years (51.9% vs. 7.6%), whereas low birthweight (1500–2499 g) predominated among those aged 35–39 years (46.8% vs. 11.1%).

**Table 1:** Socio‐demographic and obstetric characteristics by maternal age group.

Characteristics	35–39 years (*n* = 357), *n* (%)	≥ 40 years (*n* = 27), *n* (%)	Total (*n* = 384), *n* (%)	*p* value
Education				0.111
No education	74/357 (20.7)	11/27 (40.7)	85/384 (22.1)	
Primary	246/357 (68.9)	14/27 (51.9)	260/384 (67.7)	
Secondary	33/357 (9.2)	2/27 (7.4)	35/384 (9.1)	
Higher	4/357 (1.1)	0/27 (0.0)	4/384 (1.0)	
Working status				0.684
Not working	322/357 (90.2)	25/27 (92.6)	347/384 (90.4)	
Working	35/357 (9.8)	2/27 (7.4)	37/384 (9.6)	
Household income				0.089
≤ 15,000 BDT	113/357 (31.7)	13/27 (48.1)	126/384 (32.8)	
15,001–20,000 BDT	153/357 (42.9)	6/27 (22.2)	159/384 (41.4)	
> 20,000 BDT	91/357 (25.5)	8/27 (29.6)	99/384 (25.8)	
Any comorbidity				0.154
No	156/357 (43.7)	8/27 (29.6)	164/384 (42.7)	
Yes	201/357 (56.3)	19/27 (70.4)	220/384 (57.3)	
Nutritional status				0.334
Poor	9/357 (2.5)	2/27 (7.4)	11/384 (2.9)	
Moderate	79/357 (22.1)	8/27 (29.6)	87/384 (22.7)	
Good	266/357 (74.5)	17/27 (63.0)	283/384 (73.7)	
Excellent	3/357 (0.8)	0/27 (0.0)	3/384 (0.8)	
Physical activity				< 0.001
Sometimes	4/357 (1.1)	7/27 (25.9)	11/384 (2.9)	
Occasionally	284/357 (79.6)	14/27 (51.9)	298/384 (77.6)	
Regularly	69/357 (19.3)	6/27 (22.2)	75/384 (19.5)	
Family support				0.171
No	36/357 (10.1)	5/27 (18.5)	41/384 (10.7)	
Yes	321/357 (89.9)	22/27 (81.5)	343/384 (89.3)	
BMI category				0.610
Normal (18.5–22.9)	12/357 (3.4)	0/27 (0.0)	12/384 (3.1)	
Overweight (23.0–27.4)	133/357 (37.3)	11/27 (40.7)	144/384 (37.5)	
Obese (≥ 27.5)	212/357 (59.4)	16/27 (59.3)	228/384 (59.4)	
ANC visits				0.429
Inadequate (0–3)	26/357 (7.3)	2/27 (7.4)	28/384 (7.3)	
Partial (4–7)	239/357 (67.0)	15/27 (55.6)	254/384 (66.2)	
Adequate (≥ 8)	92/357 (25.8)	10/27 (37.0)	102/384 (26.6)	
Parity				0.453
1 (Primipara)	31/357 (8.7)	1/27 (3.7)	32/384 (8.3)	
2–4 (Multipara)	317/357 (88.8)	26/27 (96.3)	343/384 (89.3)	
≥ 5 (Grand multipara)	9/357 (2.5)	0/27 (0.0)	9/384 (2.3)	
Gravida				< 0.001
1–2	25/357 (7.0)	1/27 (3.7)	26/384 (6.8)	
3–4	197/357 (55.2)	5/27 (18.5)	202/384 (52.6)	
≥ 5	135/357 (37.8)	21/27 (77.8)	156/384 (40.6)	
Mode of delivery				0.073
NVD	60/357 (16.8)	1/27 (3.7)	61/384 (15.9)	
CS	297/357 (83.2)	26/27 (96.3)	323/384 (84.1)	
Place of delivery				0.535
Home	5/357 (1.4)	0/27 (0.0)	5/384 (1.3)	
Hospital	350/357 (98.6)	27/27 (100.0)	377/384 (98.7)	
Multiple birth				0.207
Singleton	337/357 (94.4)	27/27 (100.0)	364/384 (94.8)	
Twin	20/357 (5.6)	0/27 (0.0)	20/384 (5.2)	
Birthweight category				< 0.001
VLBW (< 1500 g)	27/357 (7.6)	14/27 (51.9)	41/384 (10.7)	
LBW (1500–2499 g)	167/357 (46.8)	3/27 (11.1)	170/384 (44.3)	
Normal (2500–3800 g)	163/357 (45.7)	10/27 (37.0)	173/384 (45.1)	

Table 2 shows a high burden of complications, with 97.7% of women experiencing at least one maternal adverse outcome, most commonly preeclampsia (27.3%), premature rupture of membranes (26.0%), and oligohydramnios (23.2%); eclampsia (16.9%) and postpartum hemorrhage (15.1%) were also frequent, and maternal mortality was rare (0.5%). Neonatal adverse outcomes occurred in 80.5% of births, dominated by prematurity (72.9%) and preterm delivery (74.0%), followed by low birthweight (52.3%), stillbirth (12.3%), and acute respiratory distress syndrome (22.9%).

**Table 2:** Maternal and neonatal complications among delivered women (*n* = 384).

Complication	*n*/*N* (%)	95% CI
Maternal complications		
Antepartum hemorrhage (APH)	53/384 (13.8)	10.5–17.7
Hyperemesis gravidarum	80/384 (20.8)	16.9–25.2
Premature rupture of membranes (PROM)	100/384 (26.0)	21.7–30.7
Postpartum hemorrhage (PPH)	58/384 (15.1)	11.7–19.1
Preeclampsia	105/384 (27.3)	22.9–32.1
Eclampsia	65/384 (16.9)	13.3–21.1
Obstructed labor	40/384 (10.4)	7.5–13.9
Oligohydramnios	89/384 (23.2)	19.0–27.7
Polyhydramnios	23/384 (6.0)	3.8–8.9
Puerperal sepsis	30/384 (7.8)	5.3–11.0
Maternal death	2/384 (0.5)	0.06–1.9
Neonatal complications		
Low birthweight (LBW)	201/384 (52.3)	47.2–57.4
Stillbirth	47/384 (12.3)	9.1–15.9
Macrosomia	0/384 (0.0)	0.0–1.0
Malpresentation	10/384 (2.6)	1.3–4.7
Meconium aspiration syndrome	23/384 (6.0)	3.8–8.9
Acute respiratory distress syndrome (ARDS)	88/384 (22.9)	18.8–27.5
Prematurity	280/384 (72.9)	68.2–77.3
Congenital malformation	5/384 (1.3)	0.4–3.0
Preterm delivery	284/384 (74.0)	69.3–78.3
Overall adverse outcomes		
Any maternal adverse outcome	375/384 (97.7)	95.6–98.9
Any neonatal adverse outcome	309/384 (80.5)	76.1–84.3

**Figure 2:**
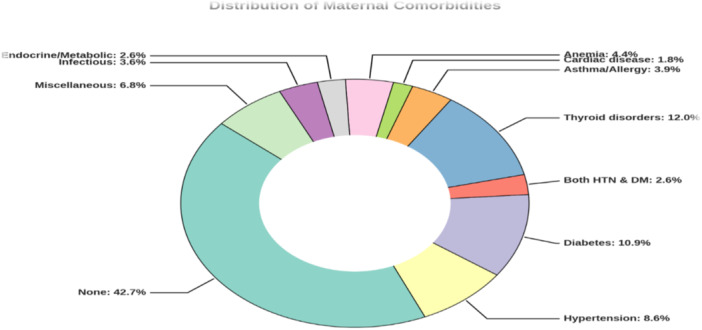
Pie chart (donut variant) showing the distribution of maternal comorbidities among delivered women. *Note:* The chart shows the proportion of women with documented pre‐existing or pregnancy‐related comorbidities at delivery. Conditions included thyroid disease, diabetes, hypertension, anemia, asthma or allergy, infectious, endocrine/metabolic, cardiac, and other disorders; women without documented conditions were classified as having no comorbidity. Percentages are based on the total sample, with mutually exclusive categories.

Figure 2 shows the distribution of maternal comorbidities. Overall, 42.7% of women had no documented pre‐existing condition. Among those with comorbidities, thyroid disorders (12.0%), diabetes (10.9%), and hypertension (8.6%) were most frequent; other conditions were less common, including anemia (4.4%), asthma or allergy (3.9%), infectious diseases (3.6%), endocrine or metabolic disorders (2.6%), cardiac disease (1.8%), combined hypertension and diabetes (2.6%), and miscellaneous conditions (6.8%).

Table 3 presents the key unadjusted and adjusted associations with maternal and neonatal adverse outcomes. In bivariate analyses, maternal adverse outcomes were nearly universal across age groups and were therefore not meaningfully differentiated by age. Maternal complications were significantly more frequent among women with comorbidities than among those without (99.5% vs. 95.1%; *p* = 0.005). Birthweight showed complete separation, with all very low and low birthweight deliveries experiencing maternal adverse outcomes.

**Table 3:** Key unadjusted and adjusted associations with maternal and neonatal adverse outcomes.

Characteristics	Maternal adverse outcome *n*/*N* (%)	Bivariate *p*	Maternal AOR (95% CI)	Adjusted *p*	Neonatal adverse outcome *n*/*N* (%)	Bivariate *p*	Neonatal AOR (95% CI)	Adjusted *p*
Age group (years)								
35–39	349/357 (97.8)	0.63	Ref	—	284/357 (79.6)	0.10	Ref	—
≥ 40	26/27 (96.3)		0.00 (0.00–1.13)	0.05	25/27 (92.6)		1.02 (0.19–5.48)	0.99
Any comorbidity								
No	156/164 (95.1)	0.005	Ref	—	119/164 (72.6)	< 0.001	Ref	—
Yes	219/220 (99.5)		12.18 (0.37–400.2)	0.16	190/220 (86.4)		1.71 (0.82–3.58)	0.15
Parity								
1	32/32 (100.0)	0.58	Ref	—	31/32 (96.9)	0.01	Ref	—
2–4	334/343 (97.4)		3.11 (0.11–90.97)	0.51	273/343 (79.6)		0.29 (0.03–2.41)	0.25
≥ 5	9/9 (100.0)		0.00 (0.00–12.55)	0.15	5/9 (55.6)		0.04 (0.00–0.86)	0.04
Birthweight								
VLBW (< 1500 g)	41/41 (100.0)	—	Ref	—	41/41 (100.0)	< 0.001	Ref	—
LBW (1500–2499 g)	170/170 (100.0)		1.82 (0.07–49.40)	0.72	169/170 (99.4)		2.45 (0.11–55.22)	0.57
Normal (≥ 2500 g)	164/173 (94.8)		0.14 (0.01–3.79)	0.25	99/173 (57.2)		0.03 (0.01–0.35)	0.006
BMI (kg/m²)								
Normal (18.5–22.9)	12/12 (100.0)	0.81	Ref	—	6/12 (50.0)	< 0.001	Ref	—
Overweight (23.0–27.4)	141/144 (97.9)		5.08 (0.05–488.9)	0.49	108/144 (75.0)		1.04 (0.20–5.34)	0.96
Obese (≥ 27.5)	222/228 (97.4)		1.57 (0.03–89.35)	0.83	195/228 (85.5)		2.60 (0.51–13.37)	0.25

*Note:* Values are presented as number/total (*n*/*N*) with percentage (%), or as adjusted odds ratios (AORs) with 95% confidence intervals. Bivariate *p* values were obtained using two‐sided Pearson *χ*² tests. Adjusted estimates were derived from Firth penalized logistic regression to address sparse data and separation. Interpretation emphasizes effect size and precision.

For neonatal outcomes, several significant associations were observed in bivariate analyses. Adverse neonatal outcomes were more common among infants born to mothers with comorbidities than among those without (86.4% vs. 72.6%; *p* < 0.001). Parity was associated with neonatal outcomes, with lower proportions of adverse outcomes among births to grand multiparous women compared with primiparous women (*p* = 0.01). Birthweight was the strongest determinant, with all very low birthweight infants and nearly all low birthweight infants experiencing neonatal complications, compared with just over half of normal‐birthweight infants (*p* < 0.001). Neonatal adverse outcomes were also more frequent among infants born to obese mothers than among those born to mothers with normal BMI (*p* < 0.001).

After adjustment using Firth penalized logistic regression, no covariate retained an independent association with the maternal composite outcome, reflecting the near‐ceiling prevalence of maternal complications in this tertiary‐care sample. For neonatal outcomes, grand multiparity remained associated with lower odds of adverse outcomes compared with primiparity (AOR 0.04; 95% CI 0.00–0.86), and normal birthweight remained strongly protective relative to very low birthweight (AOR 0.03; 95% CI 0.01–0.35). Model diagnostics are presented to characterize model behavior under separation rather than to imply predictive performance, given the high prevalence of outcomes. Full results for all covariates are provided in Table S1.

2.11

To assess potential overadjustment arising from the inclusion of a downstream clinical indicator and to evaluate robustness of the multivariable models, sensitivity analyses were conducted in which birthweight was excluded from the covariate set, and models were re‐estimated. The resulting estimates were broadly consistent with the primary analyses in both direction and overall magnitude across predictors for maternal and neonatal outcomes, with no substantive changes in key associations or statistical interpretation. These findings indicate that inclusion of birthweight did not materially influence effect estimates and that the observed relationships were not driven by model specification, supporting the stability and internal consistency of the reported results Table S2.

## 3 Discussion

This study characterizes maternal and neonatal complications among women aged ≥ 35 years delivering in tertiary referral hospitals in Dhaka and examines their co‐occurrence with sociodemographic and clinical factors within the same delivery episode. In this referral context, women aged ≥ 40 years were more frequently associated with very‐low‐birthweight deliveries, while hypertensive and membrane‐related disorders dominated the maternal case mix. Neonatal morbidity clustered around prematurity and low birthweight, with birthweight remaining the only factor consistently associated with neonatal adverse outcomes after adjustment. These findings reflect associations within a high‐risk referral population rather than causal effects.

The observed clustering of advanced maternal age, comorbidities, and neonatal vulnerability is biologically plausible and consistent with prior literature on age‐related vascular and metabolic changes linked to placental dysfunction and impaired fetal growth [34]. However, the cross‐sectional, hospital‐based design precludes causal inference and population‐level risk estimation. Instead, the findings delineate how maternal age, comorbidity profiles, and neonatal risk intersect at the point of care in tertiary facilities, with women aged ≥ 40 years representing a clinically distinct subgroup reflecting a steeper risk gradient in referral practice.

Neonatal findings are best understood in this descriptive framework. Birthweight appears as a strong marker of neonatal vulnerability, summarizing multiple upstream processes including gestational duration, maternal health status, and antenatal care exposure. Because birthweight is proximal to neonatal outcomes and part of the composite endpoint, its association should not be interpreted etiologically. Instead, it highlights how neonatal risk is distributed within the referral case‐mix and identifies strata that require heightened clinical attention during the immediate perinatal period. Similar interpretations of birthweight as a summary marker of neonatal vulnerability rather than a causal exposure have been emphasized in prior perinatal epidemiology and neonatal outcomes research [35, 36, 37].

Socioeconomic and household factors showed associations with adverse outcomes in bivariate analyses, suggesting that care‐seeking constraints, limited family support, and economic vulnerability may co‐occur with clinical risk among older gravidae. These patterns are consistent with previous evidence linking socioeconomic disadvantage to delayed care‐seeking, reduced rest during pregnancy, and barriers to referral compliance [38, 39]. However, such associations likely reflect contextual and behavioral constraints rather than independent causal effects. Importantly, these relationships may be amplified in tertiary hospitals, which selectively receive complicated cases, and therefore should not be extrapolated directly to community populations.

Two analytic considerations guide interpretation. First, the maternal composite outcome was highly prevalent in this referral population, reflecting the concentration of complicated cases typical of tertiary care. Under these conditions, adjusted maternal estimates primarily characterize patterns of complication burden across subgroups rather than identifying independent predictors. Maternal findings should therefore be interpreted descriptively rather than as evidence of etiologic effects. Second, the apparent protective signal at the extreme of parity in adjusted models warrants caution in a facility‐based sample; survivor selection and residual confounding may operate among grand multipara who reach tertiary care. By contrast, the birthweight signal is consistent across analyses and should anchor neonatal risk stratification and service preparation.

Policy translation is direct and measurable. Guidelines may consider explicitly flagging ≥ 40 years as a high‐complexity group within advanced maternal age, given the clustering of comorbidities and adverse outcomes observed in tertiary care. Facility readiness should ensure access to magnesium sulfate, antihypertensives, antenatal corticosteroids, and competent newborn respiratory support. Monitoring should track coverage of the AMA bundle among women ≥ 35 by 14 weeks, repeat growth assessments by 28 and 34 weeks, and post‐delivery small‐baby care processes. Framed this way, the discussion moves beyond description to an implementable agenda: age‐attuned antenatal care, comorbidity‐aware clinical pathways, and birthweight‐sensitive newborn care. In a metropolitan referral environment such as Dhaka's, these are pragmatic levers that speak directly to the risk architecture revealed by the data and offer a defensible route to reduce preventable morbidity at the older end of the maternal‐age spectrum.

## 4 Strengths, Limitations, and Future Directions

This study's strengths enhance confidence in its signals and their relevance for service planning. Measurement was anchored at discharge, so exposures and outcomes were captured at a uniform clinical point and verified against standardized obstetric and neonatal records. Consecutive recruitment across two high‐volume referral hospitals over 7 months achieved the prespecified sample (*n* = 384). Data quality was reinforced through trained interviewers, same‐day supervisory checks, de‐duplication, and an independent faculty audit. Variable definitions mirrored routine documentation, improving fidelity and clinical interpretability. Analytically, composite endpoints limited multiple testing in the presence of rare events, and penalized logistic regression appropriately addressed sparse cells; the neonatal model's strong discrimination supports the robustness of the birthweight signal.

Important limitations should be considered. The cross‐sectional design and restriction to women aged ≥ 35 years preclude causal inference, internal comparisons with younger mothers, and population‐level estimation of risk attributable to maternal age. The hospital‐based tertiary setting concentrates high‐risk pregnancies and limits generalizability to community or primary‐care contexts. Enrollment at discharge may have underrepresented women who were clinically unstable or had incomplete records, potentially leading to an underestimation of severe maternal and neonatal complications. Outcomes were captured during hospitalization only; post‐discharge maternal events and late neonatal morbidity were not assessed.

The maternal composite outcome was broad and approached a ceiling, producing separation and unstable adjusted estimates; maternal contrasts should therefore be interpreted descriptively rather than as independent predictors. Although penalized regression mitigated sparse‐data bias, estimates may remain sensitive to model specification and sample size. Because outcome prevalence was high, odds ratios may overestimate relative risks and should be interpreted cautiously as measures of association rather than causal effect size. Some variables (e.g., physical activity, family support) were self‐reported, and several constructs (ANC adequacy, nutritional status, income, parity, gravidity) were categorized to match routine hospital documentation, which may have reduced precision, obscured gradients, and introduced residual confounding within categories. In addition, certain clinical severity indicators, including timing of antenatal care initiation, blood pressure levels, and gestational age, were not consistently available as continuous measurements in hospital records, limiting assessment of dose–response relationships.

Comorbidities were aggregated as any versus none, which may mask heterogeneity in type, severity, and timing. Missing data patterns were not formally modeled; although exclusions were minimal, complete‐case analysis may still introduce bias if missingness was not random. Data were obtained from two hospitals, and although facility practices were similar, clustering effects cannot be fully excluded. The neonatal composite outcome combined mortality and morbidity indicators, which may obscure outcome‐specific associations. Residual confounding is possible due to unavailable factors such as pre‐pregnancy BMI, gestational weight gain, dietary intake, psychosocial stress, and quality of antenatal care. The ≥ 40‐year subgroup was small, widening confidence intervals, and apparent protective signals among grand multipara should be interpreted cautiously, given potential survivor and selection bias. Finally, gestational age–standardized anthropometry was unavailable, limiting the separation of fetal growth restriction from prematurity.

Future work should be tightly focused on two fronts. First, a prospective multi‐site cohort spanning primary and tertiary facilities with early gestational dating, weight‐for‐gestational‐age *z*‐scores, and severity‐graded maternal endpoints would reduce separation, improve generalizability, and enable stronger causal insight. Second, a pragmatic implementation study of an age‐attuned “advanced maternal age bundle” for women ≥ 35 years early screening for hypertension, diabetes, and thyroid disease; first‐trimester dating; scheduled growth surveillance; pre‐agreed escalation for hypertensive disease or threatened preterm birth paired with reliable small‐baby care (antenatal corticosteroids, thermal care, kangaroo mother care, and respiratory support) should be evaluated for coverage, timeliness, and neonatal outcomes. A brief qualitative inquiry embedded within that implementation can map practical barriers (transport, decision‐making, workload) and inform low‐cost supports without delaying scale‐up.

## 5 Conclusion

In this hospital‐based analysis of women aged ≥ 35 years, we observed a high burden of maternal complications and neonatal morbidity in a tertiary referral setting, with very‐low and low birthweight anchoring the neonatal risk profile and hypertensive and membrane‐related disorders prominent on the maternal side. After adjustment, birthweight remained the only variable consistently associated with neonatal adverse outcomes, while no covariates retained independent associations for the maternal composite outcome, reflecting the near‐ceiling prevalence of maternal events in this referral population. Although biological pathways linking advanced maternal age, comorbidities, and adverse perinatal outcomes are biologically plausible and supported by prior literature, such mechanisms were not directly measured in this study and should therefore be considered hypothesis‐generating rather than confirmatory. Accordingly, our findings are best interpreted as describing patterns of co‐occurrence and clinical vulnerability at delivery rather than etiologic mechanisms.

These results support age‐attuned antenatal care and readiness for small‐baby care in tertiary settings, including targeted screening for common comorbidities and timely identification of fetal growth restriction, while recognizing the limits of a cross‐sectional, referral‐center sample for causal inference or population‐level risk estimation. Aligning existing guidelines, referral pathways, and neonatal capacity to these observed risk patterns offers a pragmatic route to reducing preventable morbidity among older gravidae in metropolitan Bangladesh.

## Author Contributions

Conceptualization by Humayra Afroz Pinky, MD Nahid Hassan Nishan, and Gazi Md. Ehsanul Alam. Data curation and formal analysis by MD Nahid Hassan Nishan. Methodology by MD Nahid Hassan Nishan and Humayra Afroz Pinky. Writing – original draft and writing – review and editing by all authors.

## Funding

The authors have nothing to report.

## Conflicts of Interest

The authors declare no conflicts of interest.

## Transparency Statement

The corresponding author, Nahid Hassan Nishan, affirms that this manuscript is an honest, accurate, and transparent account of the study being reported; that no important aspects of the study have been omitted; and that any discrepancies from the study as planned (and, if relevant, registered) have been explained.

## Supporting information

Supporting Figure 1.

Supporting Table 1.

Supporting Table 2.

## Data Availability

The data that support the findings of this study are available on request from the corresponding author. The data are not publicly available due to privacy or ethical restrictions.
