# Supplementary material for: Adverse Maternal and Neonatal Outcomes Among Women of Advanced Maternal Age in a Tertiary‐Care Setting in Bangladesh: A Cross‐Sectional Study
Source: Health Sci Rep. 2026 Apr 22;9(4):e72424. doi: 10.1002/hsr2.72424 (PMC13103280; doi:10.1002/hsr2.72424)
Supplement: Supplementary file 1 — Supporting Figure 1. [file HSR2-9-e72424-s001.docx]

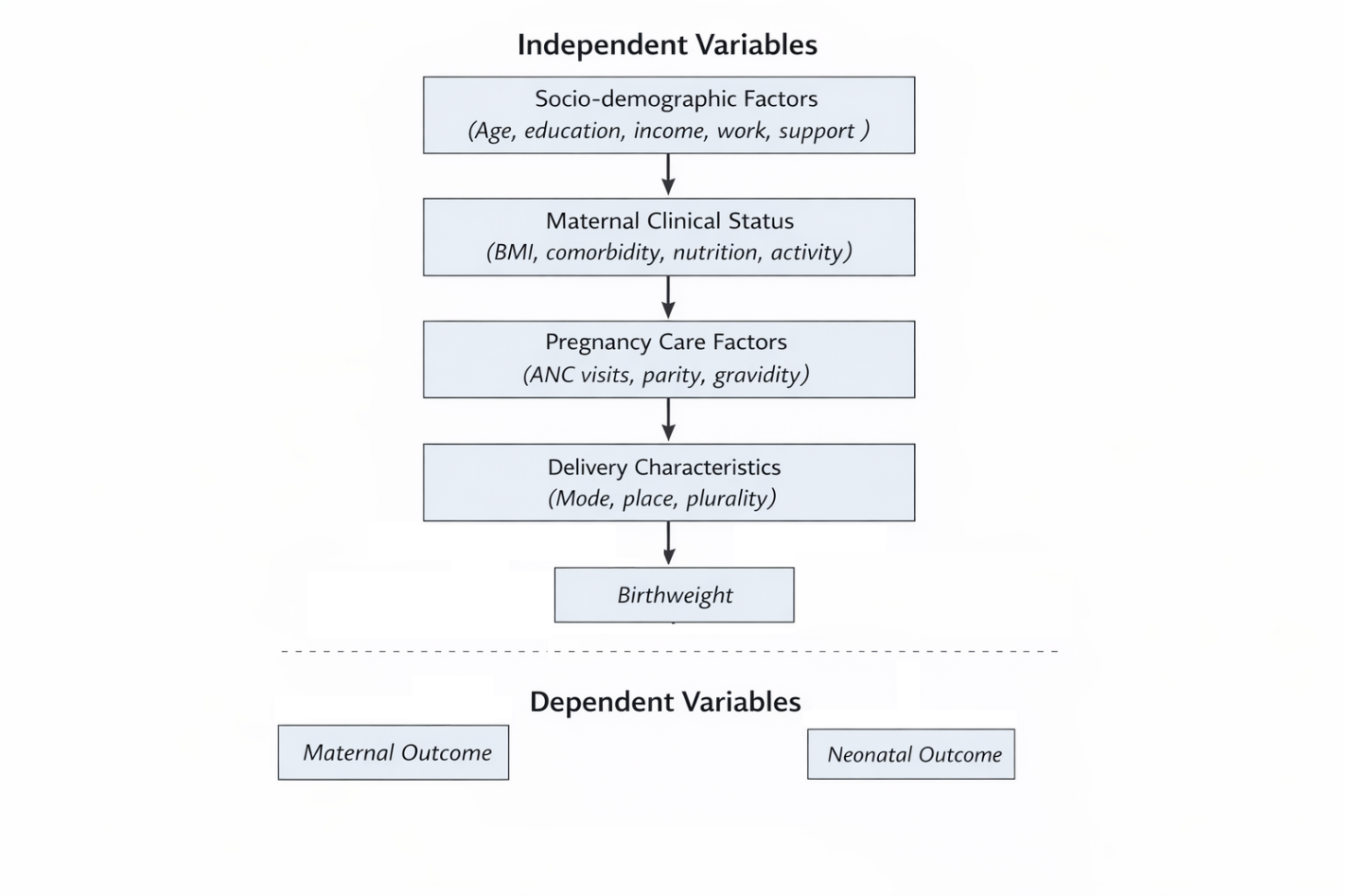


**Supplementary Figure S1:** Conceptual framework guiding covariate selection.

**Denote**: The diagram illustrates relationships among background, clinical, pregnancy-related, and delivery factors and their associations with maternal and neonatal outcomes.
