# Supplementary material for: Adverse Maternal and Neonatal Outcomes Among Women of Advanced Maternal Age in a Tertiary‐Care Setting in Bangladesh: A Cross‐Sectional Study
Source: Health Sci Rep. 2026 Apr 22;9(4):e72424. doi: 10.1002/hsr2.72424 (PMC13103280; doi:10.1002/hsr2.72424)
Supplement: Supplementary file 2 — Supporting Table 1. [file HSR2-9-e72424-s002.docx]

Supplementary Table 1: Complete bivariate and multivariable associations with maternal and neonatal adverse outcomes among women of advanced maternal age in a tertiary-care setting in Bangladesh (N = 384)

| Variable | Maternal adverse outcome n (%) | Bivariate P | Maternal AOR (95% CI) | Adjusted P | Neonatal adverse outcome n (%) | Bivariate P | Neonatal AOR (95% CI) | Adjusted P |
| --- | --- | --- | --- | --- | --- | --- | --- | --- |
| Age group (years) |  |  |  |  |  |  |  |  |
| 35–39 | 349 (97.8) | .63 | Ref | — | 284 (79.6) | .10 | Ref | — |
| ≥40 | 26 (96.3) |  | 0.00 (0.00–1.13) | .05 | 25 (92.6) |  | 1.02 (0.19–5.48) | .99 |
| Education |  |  |  |  |  |  |  |  |
| No education | 85 (100.0) | <.001 | Ref | — | 66 (77.6) | .59 | Ref | — |
| Primary | 256 (98.5) |  | 0.52 (0.03–7.89) | .64 | 214 (82.3) |  | 1.00 (0.43–2.36) | .99 |
| Secondary | 30 (85.7) |  | 0.01 (0.00–4.76) | .14 | 26 (74.3) |  | 1.30 (0.33–5.15) | .71 |
| Higher | 4 (100.0) |  | 0.10 (0.00–74.38) | .49 | 3 (75.0) |  | 1.03 (0.03–33.15) | .99 |
| Working status |  |  |  |  |  |  |  |  |
| Not working | 343 (98.9) | <.001 | Ref | — | 284 (81.8) | .04 | Ref | — |
| Working | 32 (86.5) |  | 0.01 (0.00–1.40) | .07 | 25 (67.6) |  | 0.71 (0.19–2.68) | .61 |
| Household income (BDT) |  |  |  |  |  |  |  |  |
| ≤15,000 | 124 (98.4) | .43 | Ref | — | 111 (88.1) | .03 | Ref | — |
| 15,001–20,000 | 156 (98.1) |  | 1.14 (0.08–16.88) | .92 | 124 (77.9) |  | 0.89 (0.37–2.13) | .79 |
| >20,000 | 95 (96.0) |  | 18.02 (0.31–1048.3) | .16 | 74 (74.0) |  | 1.26 (0.43–3.72) | .67 |
| Family support |  |  |  |  |  |  |  |  |
| No | 38 (92.7) | .03 | Ref | — | 32 (78.0) | .68 | Ref | — |
| Yes | 337 (98.2) |  | 403.85 (0.48–337,537) | .08 | 277 (89.4) |  | 1.54 (0.39–6.08) | .54 |
| ANC visits |  |  |  |  |  |  |  |  |
| Inadequate (0–3) | 26 (92.9) | .22 | Ref | — | 24 (85.7) | .71 | Ref | — |
| Partial (4–7) | 249 (98.0) |  | 0.28 (0.00–22.33) | .57 | 202 (79.5) |  | 0.87 (0.18–4.24) | .86 |
| Adequate (≥8) | 100 (98.0) |  | 0.04 (0.00–6.50) | .21 | 83 (81.4) |  | 0.90 (0.17–4.79) | .90 |
| Parity |  |  |  |  |  |  |  |  |
| 1 | 32 (100.0) | .58 | Ref | — | 31 (96.9) | .01 | Ref | — |
| 2–4 | 334 (97.4) |  | 3.11 (0.11–90.97) | .51 | 273 (79.6) |  | 0.29 (0.03–2.41) | .25 |
| ≥5 | 9 (100.0) |  | 0.00 (0.00–12.55) | .15 | 5 (55.6) |  | 0.04 (0.00–0.86) | .04 |
| Birthweight |  |  |  |  |  |  |  |  |
| VLBW (<1500 g) | 41 (100.0) | — | Ref | — | 41 (100.0) | <.001 | Ref | — |
| LBW (1500–2499 g) | 170 (100.0) |  | 1.82 (0.07–49.40) | .72 | 169 (99.4) |  | 2.45 (0.11–55.22) | .57 |
| Normal (≥2500 g) | 164 (94.8) |  | 0.14 (0.01–3.79) | .25 | 99 (57.2) |  | 0.03 (0.01–0.35) | .006 |
| BMI (kg/m²) |  |  |  |  |  |  |  |  |
| Normal (18.5–22.9) | 12 (100.0) | .81 | Ref | — | 6 (50.0) | <.001 | Ref | — |
| Overweight (23.0–27.4) | 141 (97.9) |  | 5.08 (0.05–488.9) | .49 | 108 (75.0) |  | 1.04 (0.20–5.34) | .96 |
| Obese (≥27.5) | 222 (97.4) |  | 1.57 (0.03–89.35) | .83 | 195 (85.5) |  | 2.60 (0.51–13.37) | .25 |

**Footnote**

*Values are presented as n (%) or adjusted odds ratios (AORs) with 95% confidence intervals. Bivariate P values were obtained using two-sided Pearson χ² tests, and adjusted estimates were derived from Firth penalized logistic regression to address sparse data and separation. Interpretation prioritizes effect size and precision rather than hypothesis testing alone. Model diagnostics showed that the maternal outcome model had a Wald χ² of 9.92 (P = .99) and a ROC AUC of 1.00, while the neonatal outcome model had a Wald χ² of 55.35 (P < .001) and a ROC AUC of 0.93 (95% CI: 0.90–0.95).*
