# Supplementary material for: Adverse Maternal and Neonatal Outcomes Among Women of Advanced Maternal Age in a Tertiary‐Care Setting in Bangladesh: A Cross‐Sectional Study
Source: Health Sci Rep. 2026 Apr 22;9(4):e72424. doi: 10.1002/hsr2.72424 (PMC13103280; doi:10.1002/hsr2.72424)
Supplement: Supplementary file 3 — Supporting Table 2. [file HSR2-9-e72424-s003.docx]

Supplementary Table S2: Sensitivity analysis excluding birthweight

| Predictor | Maternal Outcome Adjusted OR (Primary) | Maternal Outcome Adjusted OR (No Birthweight) | Neonatal Outcome Adjusted OR (Primary) | Neonatal Outcome Adjusted OR (No Birthweight) |
| --- | --- | --- | --- | --- |
| Age ≥40 years | <0.01 (0.01) | <0.01* (0.01) | 1.02 (0.87) | 1.30 (0.94) |
| Primary education | 0.52 (0.72) | 0.33 (0.60) | 1.00 (0.44) | 1.59 (0.54) |
| Secondary education | 0.01 (0.03) | <0.01 (0.01) | 1.30 (0.91) | 1.20 (0.66) |
| Higher education | 0.10 (0.34) | 0.09 (0.37) | 1.03 (1.82) | 0.83 (1.03) |
| Working status | 0.01 (0.03) | 0.03 (0.05) | 0.71 (0.48) | 0.66 (0.34) |
| Income 15,001–20,000 BDT | 1.14 (1.57) | 0.10 (0.23) | 0.89 (0.40) | 0.46* (0.17) |
| Income >20,000 BDT | >100 (37.37) | 2.71 (4.59) | 1.26 (0.70) | 0.40* (0.18) |
| Any chronic disease | 12.18 (21.70) | 3.76 (4.36) | 1.71 (0.64) | 2.26** (0.68) |
| Moderate nutritional status | >100 | >100 | 0.74 (2.69) | 0.16 (0.24) |
| Good nutritional status | 57.24 | 8.76 | 2.71 | 0.26 |
| Excellent nutritional status | 0.13 (0.46) | <0.01 (0.01) | 0.07 (0.28) | 0.14 (0.30) |
| Occasional activity | <0.01 | <0.01 | 0.26 (0.92) | 0.29 (0.44) |
| Regular activity | <0.01 | <0.01* | 0.40 (1.42) | 0.29 (0.46) |
| Family support | >100 | 21.76 | 1.54 | 1.34 |
| ANC visits 4–7 | 0.28 | 0.18 | 0.87 | 0.78 |
| ANC visits ≥8 | 0.04 | 0.08 | 0.90 | 0.81 |
| Multipara (2–4) | 3.11 | 1.41 | 0.29 | 0.22 |
| Grand multipara (≥5) | <0.01 | 0.01 | 0.04* | 0.04** |
| Cesarean delivery | 0.69 | 0.11 | 0.70 | 1.27 |
| Twin pregnancy | 2.17 | 0.40 | 14.80 | 10.06 |
| Overweight BMI | 5.08 | 3.96 | 1.04 | 1.75 |
| Obese BMI | 1.57 | 1.32 | 2.60 | 3.89* |

**Footnote**: OR = adjusted odds ratio; SE = standard error; ANC = antenatal care; BMI = body mass index. Primary models include birthweight; sensitivity models exclude birthweight. Values <0.01 indicate extremely small estimates due to sparse data. Values >100 indicate unstable large estimates. p < 0.05, ** p < 0.01.
